# Light-induced injury in mouse embryos revealed by single-cell RNA sequencing

**DOI:** 10.1186/s40659-019-0256-1

**Published:** 2019-08-29

**Authors:** Bo Lv, Chaojie Liu, Yu Chen, Lingbin Qi, Lu Wang, Yazhong Ji, Zhigang Xue

**Affiliations:** 1 Department of Regenerative Medicine, School of Medicine, Tongji University, Shanghai, 200092 China; 2 School of Life Sciences and Environment, Avans University of Applied Sciences, Breda, 4818 AJ The Netherlands; 3 Reproductive Medicine Center, Tongji Hospital, Tongji University, Shanghai, 200065 China

**Keywords:** Light exposure, Light-induced response, In vitro culture, Single-cell RNA-seq

## Abstract

**Background:**

Light exposure is a common stress factor in in vitro manipulation of embryos in the reproductive center. Many studies have shown the deleterious effects of high-intensity light exposure in different animal embryos. However, no transcriptomic studies have explored the light-induced injury and response in preimplantation embryos.

**Results:**

Here, we adopt different time-courses and illumination intensities to treat mouse embryos at the 2-cell stage and evaluate their effects on blastulation. Meanwhile, single-cell transcriptomes from the 2-cell to blastocyst stage were analyzed after high-intensity light exposure. These data show that cells at each embryonic stage can be categorized into different light conditions. Further analyses of differentially expressed genes and GO terms revealed the light-induced injury as well as the potential repair response after high-intensity lighting. Maternal-to-zygote transition is also affected by the failure to remove maternal RNAs and deactivate zygotic genome expression.

**Conclusion:**

Our work revealed an integrated response to high-intensity lighting, involving morphological changes, long-lasting injury effects, and intracellular damage repair mechanisms.

## Background

An assisted reproduction technology (ART) procedure usually implies the exposure of embryos to visible light during embryo inspection and transportation. Since the in vivo environment of embryos is much darker than that in vitro, the exposure to light is an unnatural stress factor for embryos in in vitro fertilization (IVF) procedures [1]. This light exposure during embryo manipulation is harmful for embryo viability, as it directly induces many stress-related metabolic processes or activates reactions that lead to embryo apoptosis e.g., the generation of intracytoplasmic reactive oxygen species [2]. Previous studies have shown that high-intensity light or a long exposure time to lighting is detrimental to embryos [3–5]. However, the whole-transcriptomic changes that take place after lighting are unclear.

Mouse embryos are often considered the model for improving ART programs as a replacement for human embryos since the preimplantation progress in different mammalian embryos is similar in many ways [5–7]. In this study, we investigated the effects of different illumination intensities and exposure time-courses on preimplantation mouse embryos and further explored the transcriptomic changes under detrimental lighting through single-cell RNA sequencing.

## Results

### Effects of exposure time and light intensity on preimplantation embryos

**Fig. 1:**
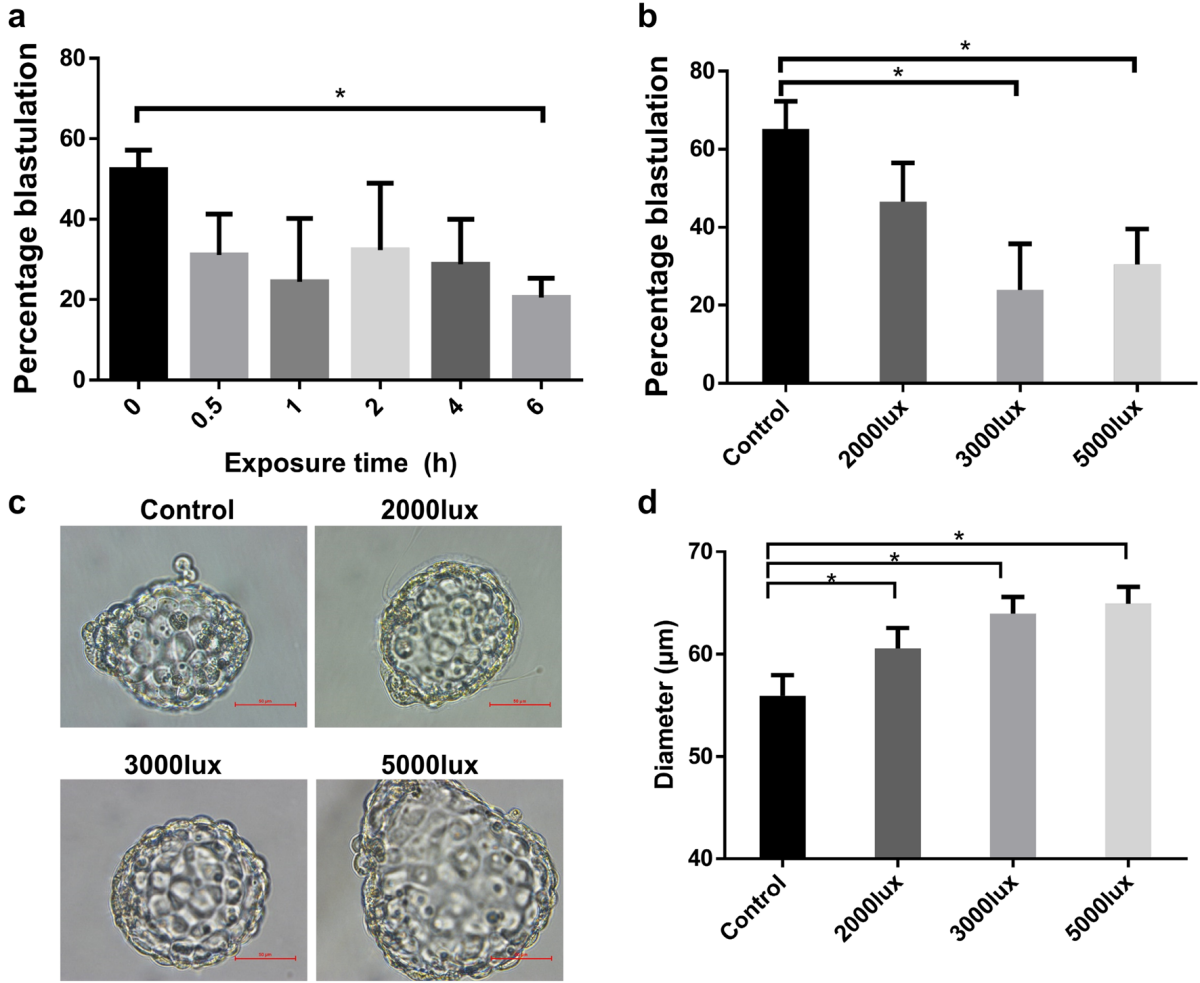
Effects of exposure time and light intensity on blastulation and morphology. **a** Blastulation percentages in different exposure time groups (total 227, 102, 98, 103, 104, and 118 embryos at the 2-cell stage after 0 h, 0.5 h, 1 h, 2 h, 4 h, and 6 h, respectively). **b** Blastulation percentages in different illumination intensity groups (total 332, 131, 152, and 191 embryos at the 2-cell stage under control, 2000 lx, 3000 lx, and 5000 lx exposure, respectively). **c** Representative images of blastocyst morphology after light exposure, scale bars = 50 μm. **d** Diameters of blastocysts after light exposure (67, 34, 47, and 52 blastocysts under control, 2000 lx, 3000 lx, and 5000 lx exposure, respectively). *p < 0.05, biological replicates ≥ 3, mean ± SE

Time control under lighting is an important factor that influences embryos’ viability in IVF centers [3]. We sought to determine the length of exposure to light for mouse preimplantation embryos. Six exposure durations from 0 to 6 h were chosen to treat embryos under the light intensity of 3000 lx. A significant decrease in the number of embryos that developed into blastocysts (20% ± 4%) was detected after 6 h exposure to light compared with that in the 0 h group (52% ± 4%) (Fig. 1a).

The light intensity is often reported in lux, and we sought to explore the effect of different light intensities on preimplantation embryos. We set three different illumination intensities (2000 lx, 3000 lx and 5000 lx) and one control group (0 lx). In the control group, the blastulation rate was 65% ± 7%, while it was significantly lower (23% ± 11% and 30% ± 9%) in the high-illumination-intensity groups (3000 lx and 5000 lx, respectively) (Fig. 1b). Intriguingly, the morphology of blastocysts after high intensity treatment was also quite different from that in the control group (Fig. 1c). Compared with the control group (55 µm), the blastocysts after light treatment (60–64 µm) were larger (Fig. 1d). Taken together, these results indicate that high light intensity might cause severe damage to preimplantation embryos and that the long exposure time was deleterious to embryonic quality.

### Single-cell transcriptome profiling of preimplantation embryos after light exposure

**Fig. 2:**
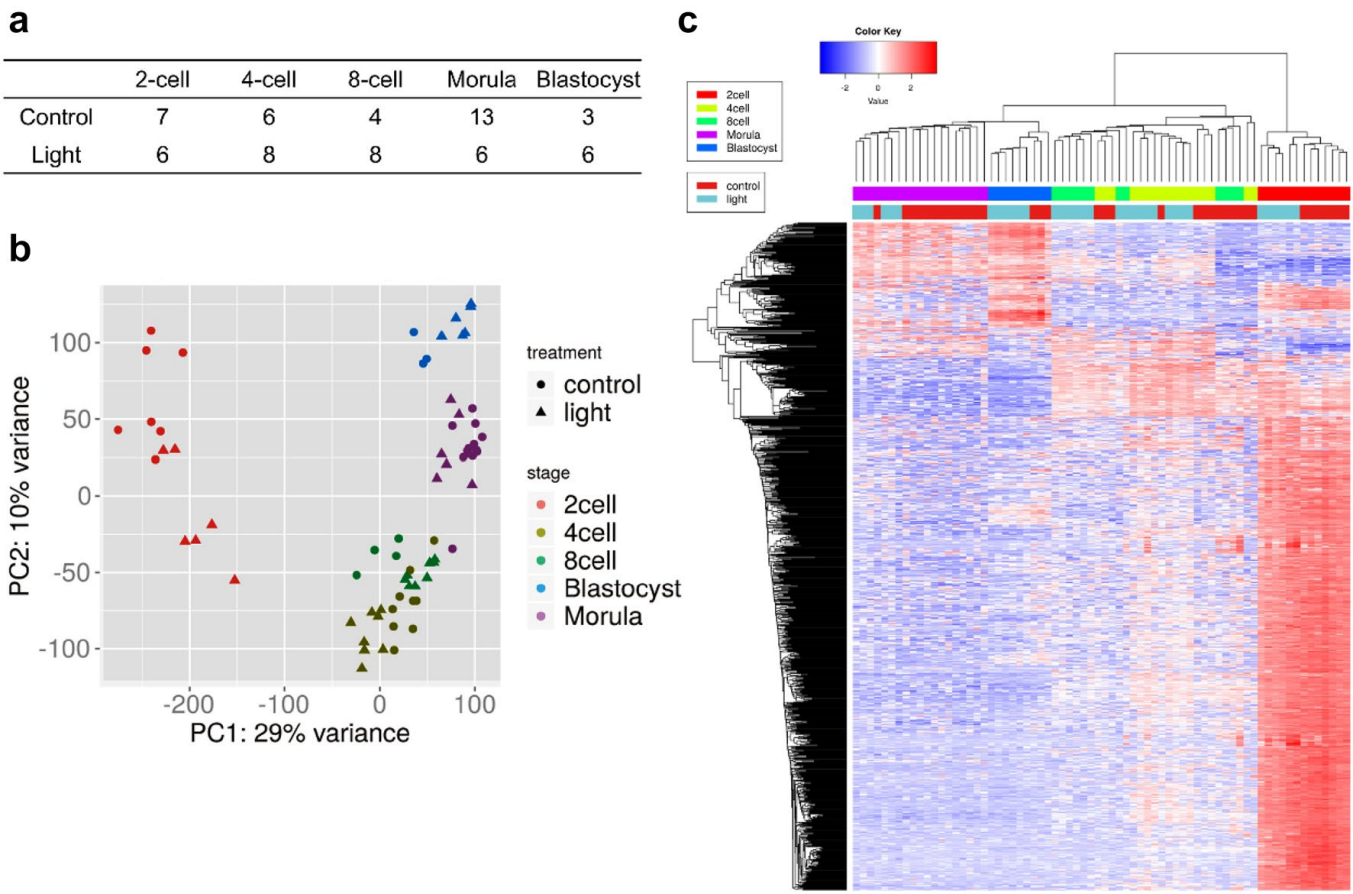
Single-cell RNA-seq transcriptome profiling of preimplantation embryos after light exposure. **a** Number of cells at each embryonic stage (2-cell to blastocyst) retained after quality filtering. **b** Two-dimensional PCA representation of single-cell transcriptomes. **c** Hierarchical clustering of highly viable genes across all cells

**Fig. 3:**
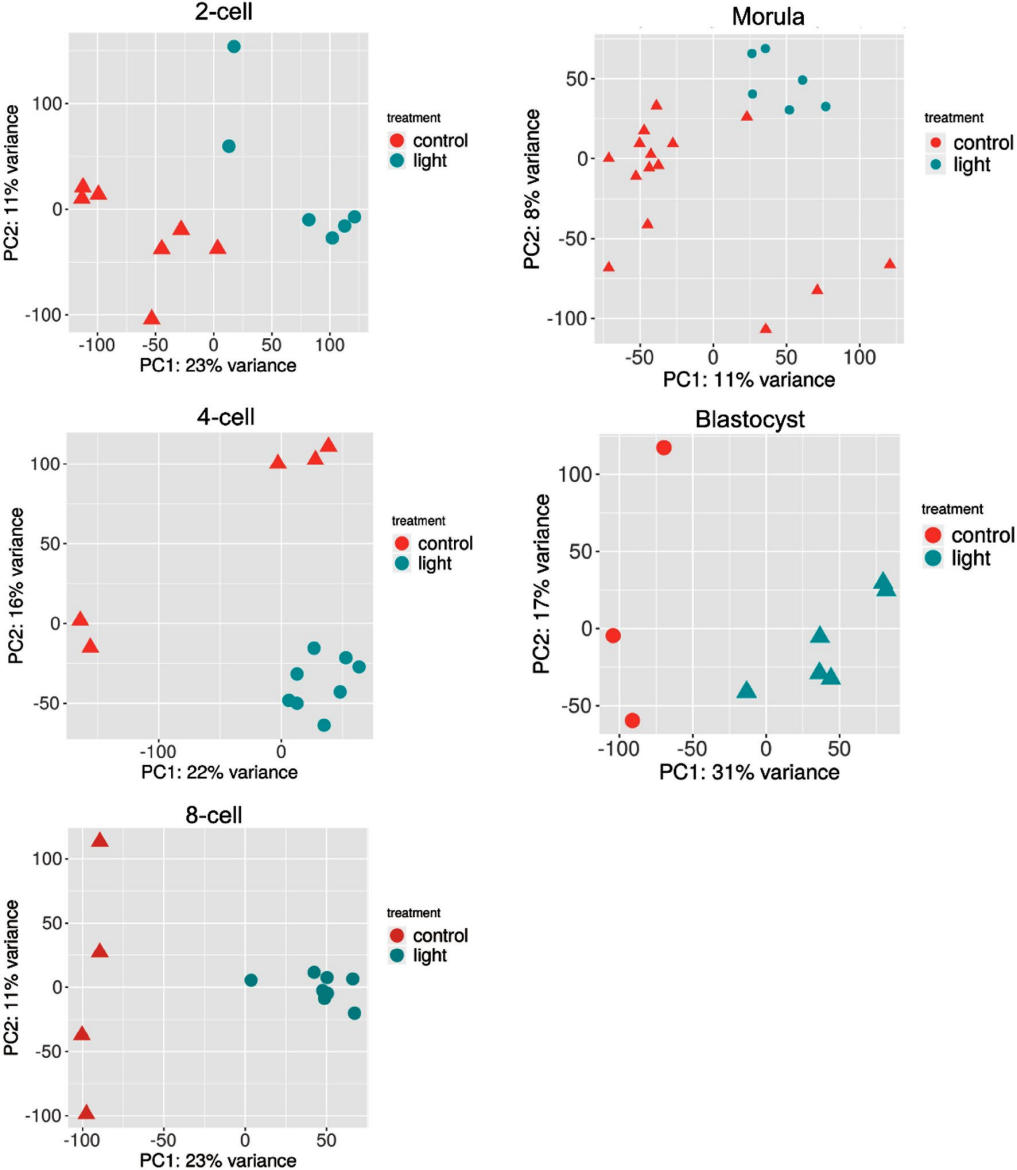
Two-dimensional PCA representation of single-cell transcriptomes at each embryonic stage

To gain insight into the response to lighting in preimplantation embryos, a total of 67 single cells of embryos from the 2-cell stage to the blastocyst stage were harvested (Fig. 2a). Principal component analysis (PCA) and unsupervised hierarchal clustering analyses revealed that cells of different preimplantation stages formed distinct clusters (Fig. 2b, c). Further analysis of PCA and heatmap data from individual stages showed differential gene expression between the control group and the light group (Fig. 3 and Additional file 1: Figure S1). Using the criteria of FDR < 0.05 and fold change ≥ 2, 8517, 4759, 6315, 5828, and 4608 differentially expressed genes (DEGs) were found in the light group compared to the control group from the 2-cell, 4-cell, 8-cell, morula and blastocyst stages, respectively. Among these genes, 4324, 2498, 3616, 2925, 672 were upregulated and 4193, 2261, 2699, 2903, and 3936 genes were downregulated from the 2-cell stage to the blastocyst stage, respectively (Additional file 1: Table S1).

### Lasting light-induced response in preimplantation embryos

**Fig. 4:**
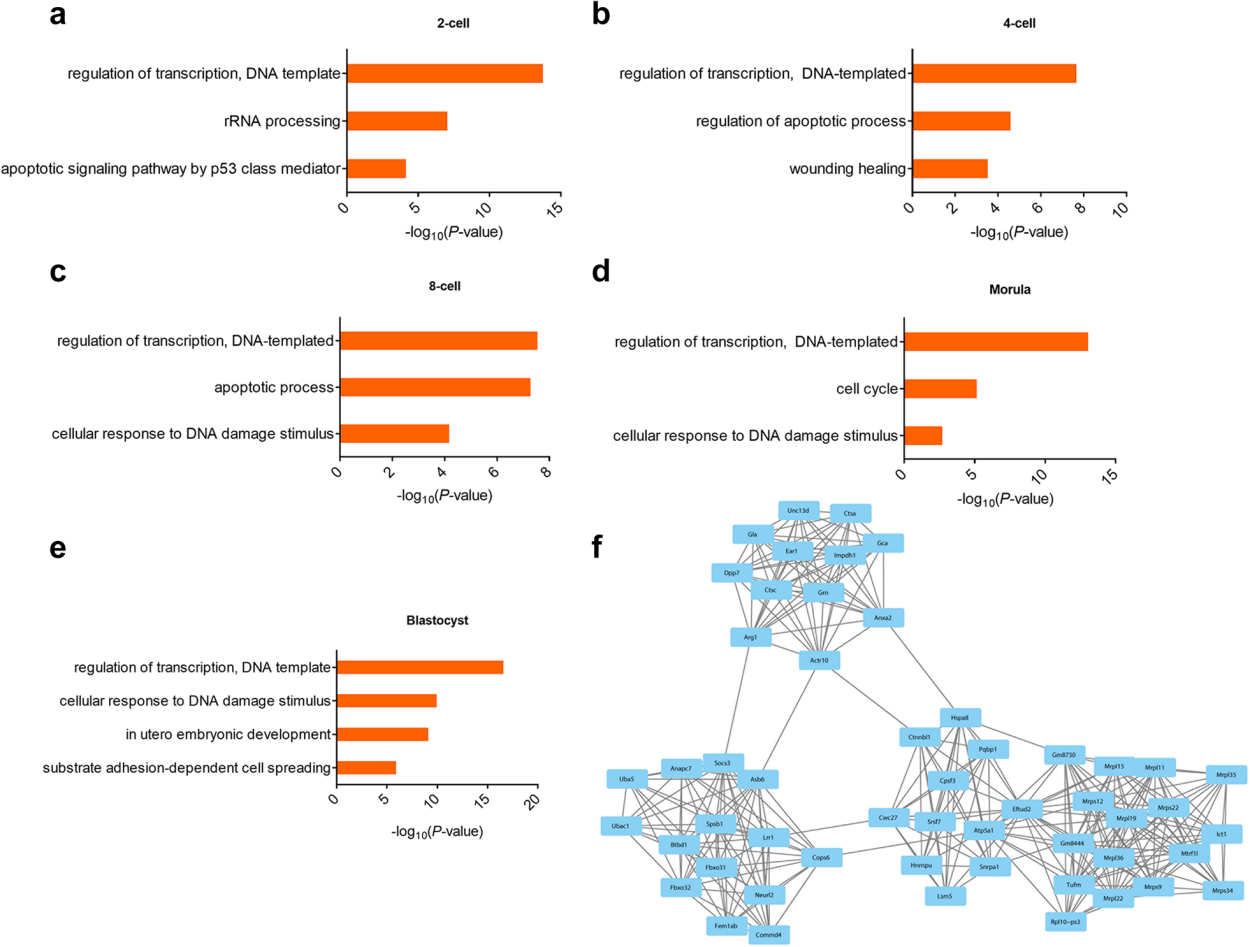
Light-induced response in preimplantation embryos. **a**–**e** GO analysis of DEGs at each embryonic stage. **f** The top module from the PPI network among upregulated DEGs at the 2-cell stage

To further elucidate the mechanism underlying the response to lighting across preimplantation embryonic stages, we performed gene ontology (GO) analysis on DEGs at each stage to investigate their biological function. The results showed preferential enrichment for biological processes in the regulation of transcription (DNA template) across all stages (Fig. 4a–e). Intriguingly, DEGs at the 4-cell to 8-cell stages were highly enriched in the apoptosis process (Fig. 4b, c). Moreover, the cell response to DNA damage was enriched from the 8-cell stage until blastocyst and in utero embryonic development, and substrate adhesion-dependent cell spreading was specifically enriched at the blastocyst stage (Fig. 4d, e). These results indicated a lasting light-induced response in preimplantation embryos with several differences at each stage.

Since embryos were exposed to light at the 2-cell stage and the number of DEGs at this stage was much higher than at other stages (Additional file 1: Table S1), we then sought to detect the immediate embryonic response after light exposure. Based on the information in the STRING database and MCODE plug-in in Cytoscape software, a total of 893 nodes and 3091 edges were identified among all upregulated genes after light exposure. The most significant module was analyzed, including 53 genes with 328 edges (Fig. 4f). The GO enrichment analysis showed that these genes were mainly associated with mitochondrial translational termination and mRNA splicing via the spliceosome (Additional file 1: Figure S2), showing the immediate and highly-relative response at the 2-cell stage after light exposure.

### The interference of maternal-to-zygotic transition in embryos exposed to light

**Fig. 5:**
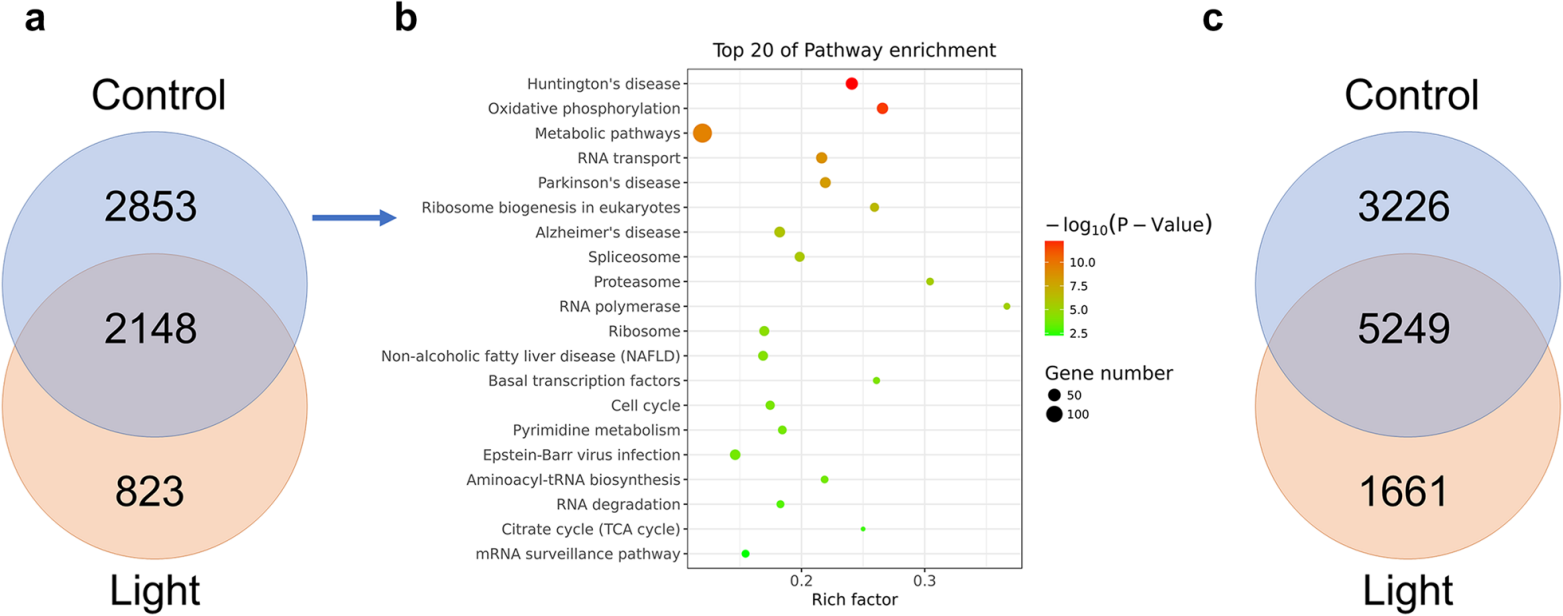
Maternal-to-zygotic transition in embryos exposed to light. **a** Venn diagram of genes upregulated at the 4-cell stage compared to the 2-cell stage in both control and light conditions; **b** bubble plot of the top 20 enriched GO terms from the analysis of the 2853 DEGs in the control group from **a**. **c** Venn diagram of genes downregulated at the 4-cell stage compared to the 2-cell stage in both control and light conditions

Zygote genome activation (ZGA), denoting the initiation of gene expression after fertilization is crucial for later embryonic development and the major ZGA wave in mice occurs between the 2-cell and 4-cell stages [8]. Therefore, DEGs of ZGA between light exposure and the control group were analyzed. A total of 57.0% (2853/5001) of the genes were not activated after light exposure (Fig. 5a). Instead, 1110 different genes were upregulated after light exposure, suggesting that the ZGA process was interfered with during high-intensity lighting. Consistent with those findings, GO analysis of the 2853 genes specifically expressed in the control group showed preferential enrichment in oxidative phosphorylation, metabolic pathways, and RNA transport (Fig. 5b), showing the dysfunction of these pathways after light exposure. The clearance of maternal mRNAs is another important event during this maternal-to-zygotic transition (MZT) [9]. Compared to the control group, high-intensity light caused the embryos to fail to downregulate 38.1% (3226/8475) of maternal RNAs at the 4-cell stage (Fig. 5c), which might also have contributed to the low blastulation rate after light exposure.

## Discussion

While undergoing the IVF procedures, the gametes, zygotes and embryos are exposed to a variable spectrum of light from different sources at the reproduction center [2]. In this study, we demonstrate the adverse effects of both high illumination intensity and long exposure time on blastulation and blastocyst morphology. Consistent with previous studies [10–12], both high intensity and long duration of lighting accelerated or accumulated the detrimental effects inside embryo cells, leading to a lower blastulation rate in our study (Fig. 1). Intriguingly, the size of blastocysts was larger after light exposure in our study, possibly resulting from the differential cell spreading events found by the GO analysis (Figs. 1d, 4e). However, these results should be carefully interpreted for human embryos, as the exposure time is quite shorter and the light intensity is lower in ART clinics than those in our study.

Single-cell RNA-seq has been widely utilized in recent years to study mammalian preimplantation embryo development [7, 13, 14]. We explored the dynamic changes in the transcriptomic profiles of preimplantation embryos from the 2-cell to the blastocyst stage after light exposure. The inclusion of almost all stages of preimplantation embryos and the analyses of this dataset revealed that cellular transcriptomic profiles after light exposure primarily segregated according to embryonic stage, followed by segregation into different light conditions (lighting or control). Our analyses also demonstrate that the damage of high-intensity and long-duration lighting to embryos existed not only at the temporal level (2-cell to blastocyst stage) but also at the spatial level (cellular morphology to intracellular transcriptome).

Light-induced injury in cells is often associated with development arrest, in which apoptosis is induced by damage to the chromosome [15–17]. Consistent with those findings, differential transcription levels, more cell apoptosis, and greater cell responses to DNA damage stimuli were found in embryos after light exposure (Fig. 4a–e). Mitochondria, which are important cell organelles, are often related to cell apoptosis and the cell cycle in many cell types [18, 19], and before the 8-cell embryonic stage, they are small spherical organelles with a dense matrix, few cristae, and a nontranscribed genome [20]. However, many hub genes encoding mitochondrial proteins were upregulated at the 2-cell stage after light exposure, e.g., *Mrpl11*, *Mrps9*, and *Mtrf1l* (Fig. 4f), suggesting abnormal mitochondrial activity and a relevant mitochondrial response to lighting. Nevertheless, hub genes *catenin beta like 1* (*Ctnnbl1*) [21] and *serine/arginine*-*rich splicing factor 7* (*Srsf7*) [22], contribute to the important functions in the spliceosome, which is required for the response to DNA damage [23]. In addition, the number of DEGs showed a peak immediately after light exposure at the 2-cell stage and then decreased (Additional file 1: Table S1). We therefore suggested a potential mechanism by which embryos to respond to and repair the injury induced by light exposure. Although these damage responses were characterized only in the blastocyst stage, further studies should be performed to explore whether the differences in the offspring or embryonic mechanisms could gradually rectify the light-induced differences beyond the blastocyst stage.

MZT is the first major developmental transition in mammalian embryos, when the zygotic genome begins transcription and maternal mRNAs are degraded [24]. Interfering with MZT might result in cell cycle arrest and finally lead to cell apoptosis or developmental delay [25, 26]. In addition to analyses of zygotic genes that were activated in the control group (Fig. 5a, b), we also categorized the genes that were activated after light exposure. Many of the top GO terms different from those in Fig. 5b were related to stress response pathways, such as ubiquitin-mediated proteolysis, base excision repair, and the MAPK signaling pathway (Additional file 1: Figure S3), which further proved the injury response after light exposure. Furthermore, 3226 genes were downregulated in normal 4-cell embryos while they maintained their expression in light-exposed embryos at this stage (Fig. 5c). These results together indicated an abnormal MZT process after light exposure.

## Conclusion

Our study not only demonstrated the damage caused by high-intensity illumination at the transcriptomic level but also provided a valuable resource for understanding the light-induced embryonic response as well as the potential self-surveillance that cells employ to protect against lighting in mouse preimplantation embryos.

## Materials and methods

### Experimental animals and ethics approval

Six-week-old female and 10-week-old male C57BL/6 mice were used for this study. All animals were kept in the animal center under controlled lighting (12:12-h light–dark cycle) with sufficient food and water.

### Retrieval of oocytes and sperm

For oocyte retrieval, unmated females were injected with 5 IU PMSG (Ningbo Sansheng Pharmaceutical Co., Ltd, China) followed by injection of 5 IU HCG (Ningbo Sansheng Pharmaceutical Co., Ltd, China) 46 h later. They were killed by cervical dislocation 12 h later, and oocytes were collected from the ampulla of the fallopian tube and then transferred into pre-equilibrated G-IVF medium (Vitrol Life, 10136). For sperm retrieval, unmated males were killed by cervical dislocation, and sperm were collected from the cauda epididymidis and then capacitated for 60 min before fertilization.

### Embryo culture and single-cell isolation

Capacitated sperm were then fertilized with oocytes for 24 h, and the 2-cell embryos were transferred into pre-equilibrated cleavage medium (Cook Medical, K-SICM-20) for subsequent culture or experiment. Embryos from the 2-cell to the blastocyst stage were dissociated into single cells using 0.25% trypsin (Gibco, 25200056) for 5 min after 30 s of Tyrode’s solution (Sigma, T1788) treatment. Single embryonic cells were manually picked into a 0.2-ml PCR tube containing lysis buffer using a mouth-operated, drawn capillary pipette for single-cell transcriptome library construction.

### Conditions for lighting

A full-spectrum electric bulb (Kesilaite, E27), whose spectral composition was shown in Additional file 1: Figure S4, was used as the light source and was installed in the incubator. A luxmeter (Huayi, MS6612) was used to measure the illumination intensity. For evaluation of the effect of light intensity on embryo development, 2000, 3000, and 5000 lx were used to treat 2-cell embryos for 6 h while 0 lx (dark condition) was used as the control group. For monitoring the effects of exposure time, 0.5, 1, 2, 4, and 6 h were used to treat 2-cell embryos under 3000 lx while 0 h (dark condition) was used as the control group. For analysis of single-cell transcriptomes in individual embryos, 5000 lx was used to treat 2-cell embryos for 6 h. All embryos after light exposure were cultured in a dark incubator for subsequent analyses. The development of blastocysts was examined 4 days later. Additionally, the morphology of blastocysts was characterized under different illumination intensities.

### Single-cell RNA-seq library construction

Single-cell RNA sequencing was performed using the SMART-seq2 protocol [27] with minor modification. Briefly, a single cell was first lysed in 0.5 µl lysis buffer and RNAs were converted using Superscript III. After purification, 0.1 ng amplified cDNA was used for library construction. Libraries were then sequenced on Illumina HiSeq ×10 in paired-end, 150 bp mode.

### RNA-seq data processing

RNA-seq data were processed on the NovelBio platform (https://cloud.novelbrain.com) with the default parameters. Briefly, gene expression was normalized by transforming mapped transcript reads to reads per kilobase of transcript per million mapped reads (RPKM). Genes with RPKM > 1 were retained for analysis. DEGs with a fold change ≥ 2 and false discovery rate (FDR) value < 0.05 were defined as statistically significant. GO enrichment analysis was performed on the platform combining the R package “topGO” and the DAVID online tool (https://david.ncifcrf.gov/). Terms with p < 0.05 were considered statistically significant. For evaluation of the protein–protein interaction (PPI) information, the DEGs were mapped to the Search Tool for the Retrieval of Interacting Genes (STRING) database (version 11.0), and interactions with a combined score > 0.4 were selected. These PPI networks were constructed using Cytoscape software (version 3.6.1) followed by module screening using the plug-in Molecular Complex Detection (MCODE).

## Supplementary information

**Additional file 1: Table S1.** DEGs across all stages after light exposure compared to the control group. **Figure S1.** Hierarchical clustering of highly viable genes across all cells at each embryonic stage. **Figure S2.** GO analysis of the top module genes in Fig. 4f. **Figure S3.** Bubble plot of the top 20 enriched GO terms from the analysis of the 823 DEGs in the light group from Fig. 5a. **Figure S4.** Spectral composition of the light source, Kesilaite E27.

## Data Availability

All sequencing data generated in this study are available on Gene Expression Omnibus (GEO) with Accession number GSE128691.

## Abbreviations

ART: assisted reproduction technology
IVF: in vitro fertilization
PCA: principal component analysis
DEG: differentially expressed gene
GO: gene ontology
ZGA: zygote genome activation
MZT: maternal-to-zygotic transition
