# Supplementary material for: Light-induced injury in mouse embryos revealed by single-cell RNA sequencing
Source: Biol Res. 2019 Aug 29;52:48. doi: 10.1186/s40659-019-0256-1 (PMC6716870; doi:10.1186/s40659-019-0256-1)

**Additional data:**

**Table S1**. DEGs across all stages after light exposure compared to the control group.


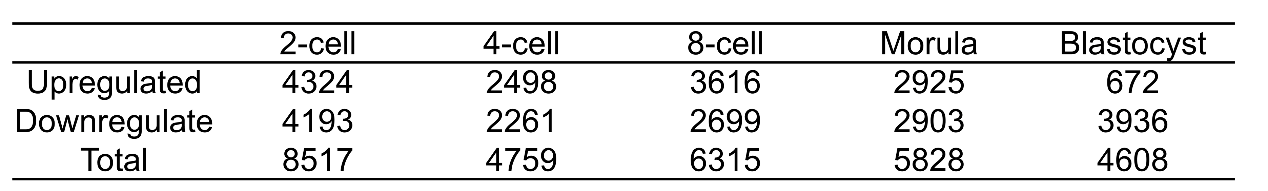


**Figure S1**. Hierarchical clustering of highly viable genes across all cells at each embryonic stage.


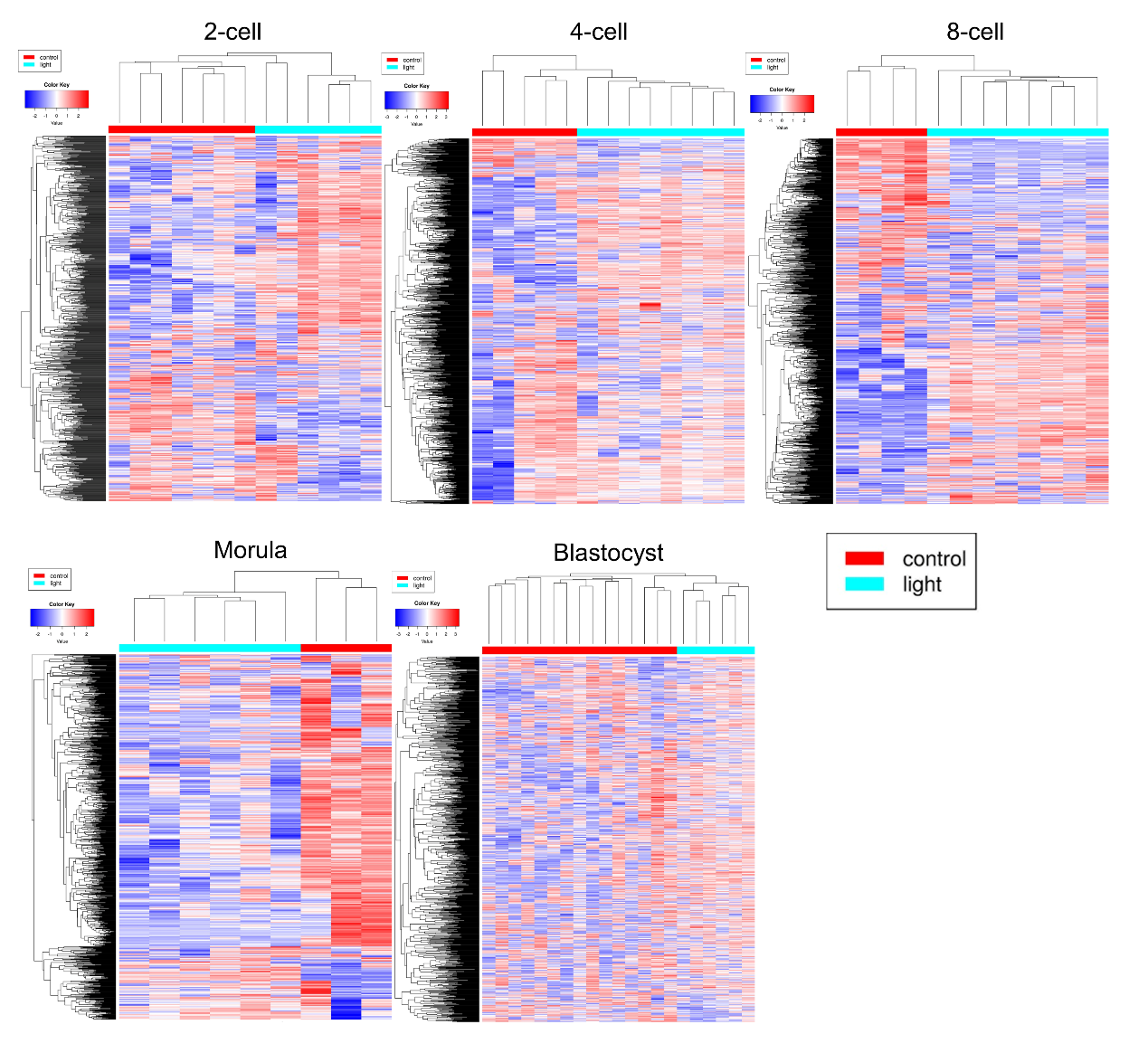


**Figure S2**. GO analysis of the top module genes in Figure 4F.


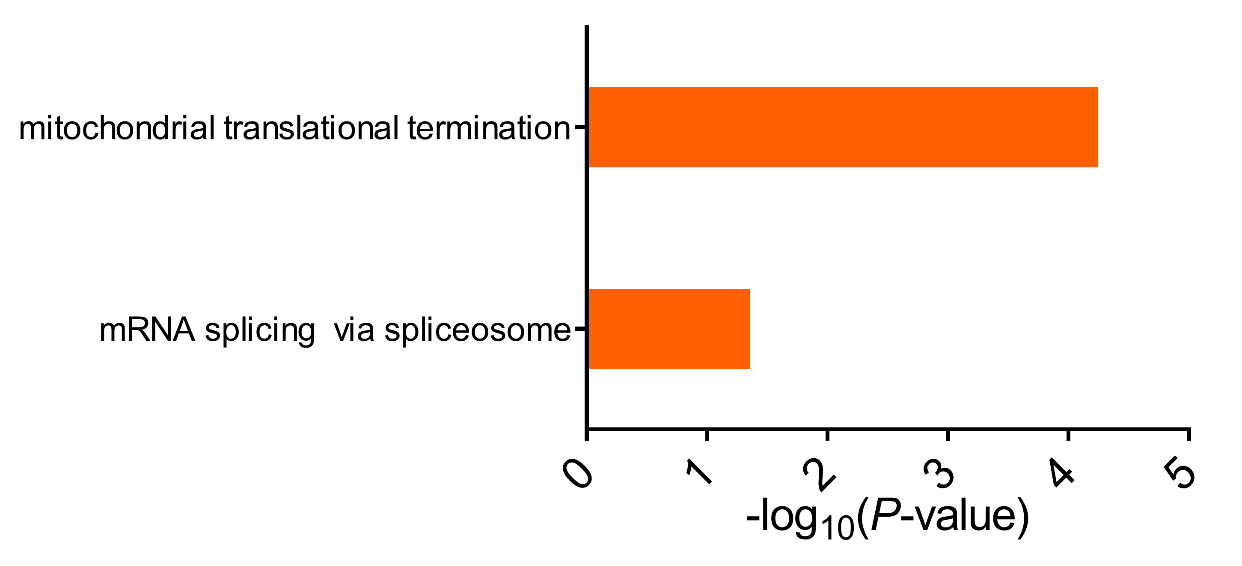


**Figure S3**. Bubble plot of the top 20 enriched GO terms from the analysis of the 823 DEGs in the light group from Figure 5A.


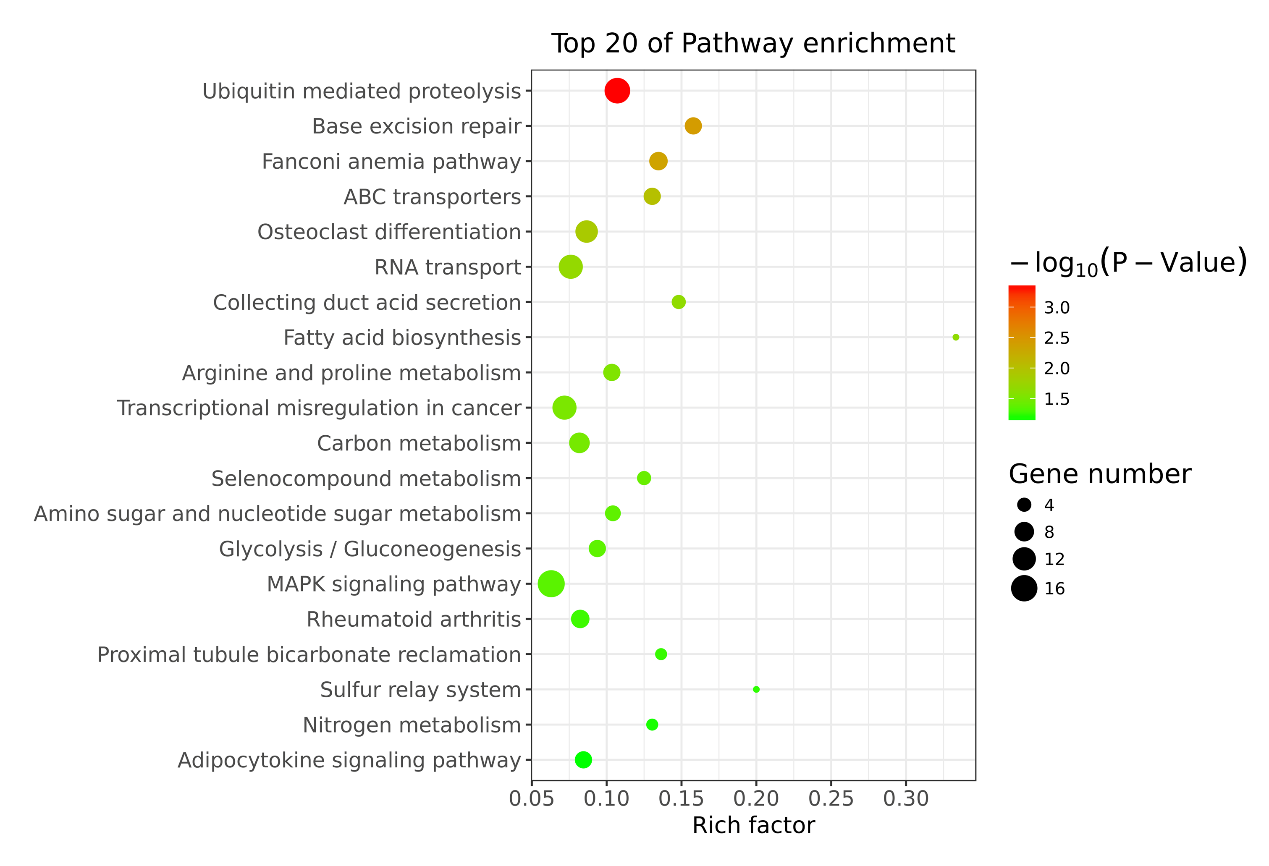


**Figure S4**. Spectral composition of the light source, Kesilaite E27.


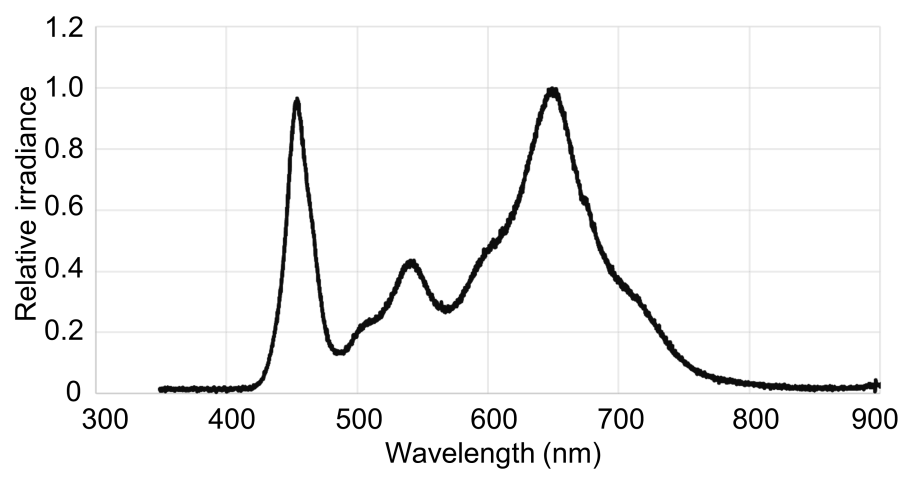

Supplement: Supplementary file 1 — Additional file 1: Table S1. DEGs across all stages after light exposure compared to the control group. Figure S1. Hierarchical clustering of highly viable genes across all cells at each embryonic stage. Figure S2. GO analysis of the top module genes in Fig. 4f. Figure S3. Bubble plot of the top 20 enriched GO terms from the analysis of the 823 DEGs in the light group from Fig. 5a. Figure S4. Spectral composition of the light source, Kesilaite E27. [file 40659_2019_256_MOESM1_ESM.docx]
